# Cases Treated at the College Clinic by Prof. Brainard

**Published:** 1858-01

**Authors:** Edwin Powell


### ARTICLE II.

### CASES TREATED AT THE COLLEGE CLINIC BY PROF. BRAINARD.

### CASE OF DOUBLE HARE-LIP—OPERATION—RECOVERY.

REPORTED BY EDWIN POWELL, M. D.

A female infant, aged ten months, a robust, healthy child, born of Irish parents, was brought to the College Clinic, Dec. 11th, 1857, with a compound complicated variety of hare-lip, viz: a fissure on each side of the mesial line, corresponding with the nostrils, which extended through the alveolar process of the upper jaw, and also the hard and soft palates. That portion of the alveolar process containing the two front incisors projected forwards nearly one inch beyond the lateral sides of the jaw.

OPERATION, *Dec.* 11, 1857.—The integument covering the projecting part having been dissected back, it was removed with the bone nippers. The copious hemorrhage from the bone, which followed its removal, was instantly and effectually checked by the application of the actual cautery. The next step in the operation was to separate the lip from the alveolar process upon each side of the fissure, so that it could easily be brought forward, and to vivify the border of the fissure, which was done by the removal of a section with a scalpel. The hemorrhage was controlled during this stage of the operation by pressure upon the facial arteries.

A leaden wire was next passed deeply through the lip, just beneath the alæ nasi, the ends of which were twisted together so as to bring the parts in contact; and afterwards two steel needles were passed through the edges of the wound, one near the inferior termination of it, and the other midway between the needle and leaden wire. Around the ends of the needles a silken ligature was passed, from one side of the division to the other, first transversly, then obliquely, in such a manner, that the thread is made to cross as many points of the wound as possible, which greatly contributes to maintaining its edges in even apposition. Lastly, the points of the needles were broken off, and the ends supported by small dossils of lint placed between them and the flesh. No other dressing was applied. The child was directed to take twelve drops of the camphorated tincture of opium every three hours.

*Dec.* 17*th.*—No unpleasant symptoms have occurred. The dressing was removed to-day, and the union of the lip was perfect. A strip of isinglass plaster was placed across the lip, to support the yet tender union. The integument which was saved from the proboscis is to make a septum nasi, which will require a second operation, after the other shall have become perfectly consolidated.

Of course, there is but one mode of treatment to be adopted in these cases; but it is an important question, concerning which there is some difference of opinion, whether the operation should be performed while the child is young, or at what time of life it should be resorted to.

Dionis, Garengeot and others advise waiting until the child is four or five years of age, on the ground that before this period the "child's cries and agitations would render the operation impracticable, or derange all the proceedings taken to insure its success." It is plain, however, that such reasons are not of great weight. A child, four or five years old, and even one eight or ten years of age, is much more difficult to manage than an infant only a few weeks old. Every child of the abovenamed age has a thousand times more dread of the pain than of the deformity, or of the inconvenience of the complaint, to which he is habituated, while an infant of tender age fears nothing, and only feels the pain of the moment.

Another class of surgeons, prominent among whom are Sir Astley Cooper, Samuel Cooper, Liston and Druitt, recommend that the operation should not be undertaken till the child is about two years of age and has cut its teeth, "because of the liability of infants of tender age to convulsions after operations." This, in fact, appears to be the only real objection against the early performance of this operation, and yet it is one which I am satisfied has been very much exaggerated, as Bell, Ledran, Mays, Roonhuysen, Pancoast, and many others plainly show.

Prof. Brainard recommended the early performance of the operation in 1837, and he has now operated on nearly one hundred and fifty cases, the greater number of which have been infants only a few weeks old, and not in a single instance have convulsions occurred. The operations have been uniformly successful, with two exceptions; one of which was a case where the fissure in the lip was caused by phagedenic ulceration, which returned in the wound after the operation; in the other, the patient died in two days after the operation, apparently from deficient nourishment.

Lawrence "thinks it very desirable to remove the defect early on every account."

The age, therefore, at which the operation may with most propriety be attempted, is from three to four weeks. The positive advantages of an operation at this period are:

*First, the absence of all fear or opposition to the performance of the operation on the part of the child.* An infant of the above-named age may, with proper care, and the administration of opiates, be kept quiet most of the time, a circumstance which favors the successful termination of the operation very much; while, on the other hand, a child one year old will scream and struggle the moment the surgeon enters the room.

*Secondly. The pressure exerted by the lip is capable, after the successful performance of the operation, of moulding the*

*alveolar process of the jaw, and of closing up the fissure extending through it and hard palate.*

*Thirdly. The ability to acquire the power of speech or articulation of sounds much more perfectly.* It is a well established fact, that the intelligence of a child is developed just in proportion as it is able to utter distinctly the sounds of language. This, then, becomes a very important consideration in deciding the time for operating, and one which ought not by any means to be lost sight of. Unless a person is able to express his ideas clearly and distinctly, they will never be of much service to himself, or any one else.

In regard to the *method* of operating, there is almost as much diversity of opinion as in regard to the time. M. Louis recommended the use of adhesive plaster, and an uniting bandage simply, believing that a perfect cure might always be accomplished by them, so as to save the patient from all the pain and annoyance of sutures. But this method of operating has been entirely abandoned, as inefficient in more than the simplest kind of cases. The twisted and interrupted sutures are the only means resorted to by surgeons of the present day; the former we believe to possess very superior advantages over the latter, for the two following reasons: *first*, the edges of the wound may be held much more perfectly and firmly in contact, and, *secondly*, the polished surface of a steel or silver needle does not ulcerate and cut through the tissues, as soon as silken ligatures, whereby the parts are held for a longer period in contact, another important consideration in cases which do not readily unite.

In those cases, where the fissure is widely separated, and where there seems to be a greater want of tissue than is common, Prof. Brainard is in the habit of using, in addition to the needles, a leaden wire passed deeply through the lips just beneath the alæ of the nose, so as to bring the fresh surfaces together by means of it. The advantage of this is that it relieves the needles of an undue amount of pressure, and also, as it does not cut through the tissues as soon as a ligature, it holds the parts in apposition for a longer time.

In the compound complicated variety of hare-lip, we often find a projection of one side of the jaw bone, in the form of a proboscis, which must be removed, as preliminary to the operation on the soft parts, by the bone nippers. It has frequently been found necessary to employ the actual cautery in these cases, to arrest the hemorrhage from the bone which follows the removal of this proboscis. It is not necessary to support the cheeks, either by an uniting bandage, or adhesive plaster, during the treatment.

The needles should be removed on the fifth or sixth day, and isinglass plaster applied to support the yet tender union.

The child should be kept as quiet as possible, for which purpose opiates may be administered.

